# Medical and Physical Intelligence

**Published:** 1821-06

**Authors:** 


### Medical and Physical Intelligence.

---

*Continuation of the Analysis of the Contents of the first Number of the Journal of Experimental Physiology of* Dr. Magendie.

"*Note on the Introduction of Viscous Liquids into the Organs of the Circulation, and on the Formation of the Fat Liver of Birds.*" By Dr. Magendie.

DR. MAGENDIE, it appears, in some experiments in which his object was to change the nature of the blood, had injected oil into the veins, thinking that "this innocent substance could circulate with the blood without inconvenience:" but this was not the case; "the animal subjected to the experiment died a few instants after the injection of an ounce of olive-oil into the jugular vein."—"On examining the organs after death, I saw (Dr. Magendie says,) that the olive-oil had plugged the extreme ramifications of the pulmonary artery, and that it had thus caused the circulation and respiration to cease, by preventing the arrival of the blood at the left side of the heart by the pulmonary veins. The oil had acted, then, as an inert, impalpable powder, suspended in water, which instantly produces death if it be injected into the jugular vein, because it obstructs the the extreme divisions of the pulmonary artery." A somewhat-thick solution of gum tragacanth "produced exactly the same phenomena as the oil."

Dr. Magendie had ascertained that substances injected into one of the branches of the vena porta, acted quite differently from what they did when introduced immediately into the general venous system. A few cubic inches of air thrown suddenly into the jugular or crural vein, kill almost instantly; whilst this fluid may be introduced, in any way whatever, into the branches of the vena porta, without "inconvenience." Dr. Magendie wished to know, then, what would be the effects of oil passed into the vena porta. Two ounces of this fluid were thrown into one of the mesenteric branches of that vein: the dog, which was the subject of the experiment, continued for several days in a stage "approaching to death,"—lying on his side, breathing with difficulty, making no movements, "not even for the natural evacuations, which seemed to be effected involuntarily." After the lapse of four days, "he began to come to himself; he took some food; and three days more were sufficient to restore him to health." The injection was repeated after the lapse of eight days, with, however, three ounces of oil. "The same symptoms (Dr. Magendie says,) showed themselves, and the animal died in the night. I opened him the next morning. The vessels contained here and there some traces of oil; but the liver especially attracted my attention: this viscus was much larger than ordinarily; it was of a pinkish pale-yellow colour; some small irregular fissures were apparent on its surface, that did not appear to be of recent origin; its colour interiorly was similar to that of its surface: the organ, in a word, showed the closest analogy with the fat liver of birds.

"This last circumstance has appeared to me to be very curious. Indeed, the fat livers are produced by forcing the animals to digest a large quantity of aliments containing but little azote. These animals having no chylous system, (as I have shown in a Memoir expressly on this subject,) all that is absorbed by the intestinal surface passes immediately to the liver; whilst in the mammalia, the drinks, and probably a small part of the chyle, alone pass through the liver; the rest of the chyle passing through the thoracic duct."

"*A Memoir on the Lymphatic Vessels of Birds*," by Dr. Magendie.

Until the year 1768, as every anatomist will remember, the existence of lymphatic vessels of the digestive organs in birds was never affirmed: it was in that year that Hewson announced his supposed discovery of them, in a letter to John Hunter, which was published in the 58th volume of the Philosophical Transactions. Hewson, on the same occasion, speaks of lymphatic vessels in the neck of birds; the discovery of which, he says, did not originate with him, but with John Hunter. Dr. Magendie says he was surprised,—on reading this paper of Hewson's about three years since,—that so able an anatomist as John Hunter should have found such vessels in the neck, and have overlooked them in the abdomen, had they really existed. This reflection, notwithstanding the apology of Hewson, induced Dr. Magendie to make some researches on this subject. He chose the *goose* for the subject of his examinations, because it was in this bird that Hewson states he had discovered them. He readily found them, on each side, in the neck; but "he was not a little surprised (he says) at not perceiving any trace of lymphatic vessels in the mesentery, notwithstanding the care and attention with which the examination was made." He sought for the *considerable plexus* which, according to Hewson, *embraces the superior mesenteric artery;* but he was not more successful: and it was in vain that he endeavoured to find the double thoracic duct. He repeated his examination a second and a third time, increasing his care and precautions; but it was useless: he always readily found the vessels in the neck, but never any trace of "chyliferous vessels, nor of the thoracic duct." He repeated his researches during digestion, but they were equally fruitless. He has since dissected above fifty birds, "of all kinds," carnivorous and others, and examined them whilst digestion was going on. The result of the whole of his observations has led him to infer that "the chyliferous vessels and the thoracic ducts do not exist in birds, and that the only traces of lymphatic vessels are seen in the neck."—"What anatomical circumstances, then, (Dr. Magendie says,) can have deceived Hewson, and led him into so serious an error as that into which he has fallen?" If it related only to the chyliferous vessels, he adds, the circumstance might be explained by supposing that Hewson had mistaken nerves for them; but this will not account for what he says about the thoracic ducts, which he states that he had even injected. There is no vessel running from the abdomen to the subclavian veins: even the azygos is absent in birds. The only structures which he thinks can have been mistaken for the thoracic ducts by Hewson, are the arterial tubes which sometimes "run from the middle part of the aorta to the pulmonary arteries, which are connected with the subclavian veins; but, as these vessels are entirely *obliterated* a few days after birth, it still remains to be known how Hewson could have injected them."

The foregoing remarks were comprised in a Memoir read to the Academy of Sciences in 1819: since that time Dr. Magendie has dissected a great number of birds, and has not only confirmed his former observations, but has also ascertained that the swan and the goose are the only birds which have a lymphatic apparatus on each side of the neck, terminated by a sort of lymphatic gland which communicates in some cases immediately with the subclavian vein.

Dr. Magendie has commenced some researches relative to the lymphatic vessels of reptiles and fish, described by Hewson and Monro. Hitherto his observations have led him to infer that these animals are entirely devoid of lymphatic vessels, and that the organs described under this name are only "sanguiferous veins." The sea-turtle, he adds, is the only one of those animals which furnishes objections to the foregoing as a general inference. He has himself injected vessels in the mesentery of this animal, which had the disposition of lymphatic vessels; but "as the mesentery was detached, it was impossible for him to follow them towards the vertebral column, and, consequently, to assure himself that they were really lymphatic vessels."

*Curious Phenomena resulting from Blood-letting in a Horse.*—Mr. Bouley, a very able veterinary surgeon of Paris, bled a horse, having pneumonia, in the neck, with the phleme, in the usual way. Nothing particular occurred during the early part of the operation; but, as the vessel into which the blood was received was not large enough to contain the quantity which Mr. Bouley wished to take, he, on this vessel being full, suspended the compression on the vein below the puncture, whilst the vessel was emptied. At the instant when the compression ceased, he heard a remarkable noise, which he had several times noticed in the course of his practice, without any ill consequence following the event, and to which he now, therefore, paid but little attention. The bleeding was completed, and the animal led into his stable. He had but just arrived there when he was affected with a general trembling; his breathing became laborious and *plaintive;* his pulse small, irregular, and much accelerated; and, finally, he uttered some deep groans, and fell down in his stall "as if stricken by lightning." On reflecting on the whole of the circumstances of the case, Mr. Bouley believed that the noise he heard, above alluded to, arose from the rushing of air into the vein; and he instantly determined to draw more blood from the animal. As the blood flowed, the horse "appeared to assume a new life:" he made some efforts to get on his legs, but did not succeed until the lapse of five or six minutes from the last bleeding. When up, his pulse became sensibly developed, lost its rapidity; his breathing became deeper; and, in half an hour from the time of the accident, he seemed to be in "the same state as before the first bleeding." Some new phenomena were now observed. The horse experienced, during the whole of the afternoon of the same day, "an extreme degree of sensibility of the whole of the right side of the body, (the side opposite to that in which the venesection was practised,) accompanied with very intense pruitus: he laid down and rolled himself about on this side, to rub himself against any objects that offered resistance."

The pneumonia run its ordinary course, and terminated favourably. Thirty days after the accident the horse was put to his ordinary work, and has not since shown any sign of disease.

This case is related in the 2d Number of Dr. Magendie's Journal, and Dr. M. remarks that Professor Dupuy, of Alfort, has mentioned to him that he had witnessed a similar accident, in which a second bleeding was also immediately effected. This case terminated favourably. Dr. Magendie doubts whether sufficient air was introduced to have proved mortal if the second blood-letting had not been resorted to. He injected some air (he does not say how much,) into a vein of a dog, and then bled him; but the animal died as soon as if he had not been bled immediately after the introduction of the air.

---

Mr. Carpue will commence his Summer Course of Lectures on the 11th of June.

### METEOROLOGICAL JOURNAL.

By Messrs. William Harris and Co. 50, Holborn, London.

*From April 20 to May 19, inclusive.*

| Day of Month. | Moon. | Rain gauge | THERM. | | | BAROM. | | DeLuc's Hygro. | | WIND. | | ATMOSPHERIC VARIATION. | | |
|---|---|---|---|---|---|---|---|---|---|---|---|---|---|---|
| Apr 20 | | ·19 | 45 | 61 | 40 | 29·57 | 29·66 | 60 | 65 | SSW | E | Rain | | Th. &c. |
| 21 | | | 44 | 60 | 39 | 29·75 | 29·87 | 55 | 56 | NNE | E | Fine | | Cloud. |
| 22 | | | 42 | 58 | 42 | 29·96 | 29·81 | 59 | 60 | E | ENE | Fine | | |
| 23 | | ·09 | 47 | 65 | 48 | 29·70 | 29·42 | 62 | 60 | E | E | Fine | | Rain |
| 24 | | | 50 | 67 | 50 | 29·53 | 29·67 | 60 | 60 | SSW | SSW | Fine | | |
| 25 | ☽ | | 55 | 69 | 58 | 29·70 | 29·77 | 57 | 60 | E | ESE | Fine | | |
| 26 | | ·07 | 63 | 71 | 57 | 29·80 | 29·70 | 58 | 56 | E | SE | Fine | | Light. |
| 27 | | | 60 | 65 | 58 | 29·72 | 29·84 | 58 | 56 | WSW | WSW | Cloudy | Fine | |
| 28 | | | 60 | 68 | 53 | 29·86 | 29·84 | 56 | 55 | ESE | E | Fine | | |
| 29 | | | 57 | 59 | 49 | 29·87 | 30·00 | 56 | 56 | ENE | NNE | Fine | | Cloud. |
| 30 | | ·02 | 52 | 56 | 48 | 30·05 | 30·08 | 56 | 57 | ENE | ENE | Cloudy | | |
| May 1 | ● | | 50 | 58 | 49 | 30·03 | 29·94 | 56 | 57 | NNE | NE | Cloudy | | Fine |
| 2 | | | 51 | 60 | 53 | 29·86 | 29·81 | 56 | 57 | NE | S | Fine | | Cloud. |
| 3 | | | 56 | 62 | 54 | 29·75 | 29·70 | 57 | 58 | SSW | E | Cloudy | Fine | Cloud. |
| 4 | | | 57 | 65 | 55 | 29·70 | 29·73 | 58 | 58 | ESE | SW | Fine | | |
| 5 | | | 59 | 68 | 50 | 29·64 | 29·51 | 58 | 57 | SW | SE | Cloudy | Fine | |
| 6 | | ·15 | 53 | 62 | 47 | 29·60 | 29·64 | 57 | 57 | SW | SSW | Fine | | Rain |
| 7 | | ·09 | 50 | 60 | 43 | 29·81 | 29·93 | 57 | 58 | SW | SSW | Fine | Sho'ry | |
| 8 | ☾ | ·26 | 50 | 63 | 48 | 29·99 | 30·11 | 57 | 57 | WSW | WSW | Rain | | |
| 9 | | | 55 | 68 | 50 | 30·17 | 30·19 | 55 | 56 | SW | SW | Fine | | |
| 10 | | | 58 | 63 | 55 | 30·25 | 30·18 | 53 | 55 | W | SW | Fine | | |
| 11 | | | 59 | 65 | 49 | 30·02 | 29·90 | 57 | 60 | WSW | W | Cloudy | Fine | |
| 12 | | | 51 | 64 | 45 | 29·87 | 29·51 | 57 | 59 | WNW | W | Fine | | |
| 13 | | ·05 | 47 | 59 | 43 | 29·43 | 29·40 | 60 | 60 | W | W | Showery | | |
| 14 | | ·03 | 48 | 57 | 41 | 29·36 | 29·33 | 59 | 57 | W | S | Fine | | Sho'ry |
| 15 | | ·15 | 49 | 56 | 42 | 29·41 | 29·52 | 57 | 55 | W | W | Cloudy | Rain | |
| 16 | | | 50 | 55 | 40 | 29·76 | 29·98 | 55 | 60 | SW | SW | Fine | Sho'ry | Fine |
| 17 | ○ | ·29 | 51 | 57 | 41 | 29·99 | 30·11 | 60 | 59 | W | NW | Rain | | |
| 18 | | ·13 | 51 | 59 | 42 | 30·00 | 30·05 | 62 | 60 | NW | NNE | Fine | Sho'ry | Rain |
| 19 | | ·02 | 49 | 52 | 42 | 30·13 | 30·20 | 60 | 57 | NW | NNE | Fine | | Sho'ry |

The quantity of rain fallen in the month of April, is 1 inch and 67-100ths.

